# Polymer microchamber arrays for geometry-controlled drug release: a functional study in human cells of neuronal phenotype

**DOI:** 10.1039/c8bm01499j

**Published:** 2019-03-27

**Authors:** Olga Kopach, Kayiu Zheng, Olga A. Sindeeva, Meiyu Gai, Gleb B. Sukhorukov, Dmitri A. Rusakov

**Affiliations:** a Department of Clinical and Experimental Epilepsy , UCL Queen Square Institute of Neurology , University College London , London WC1N 3BG , UK . Email: d.rusakov@ucl.ac.uk; b School of Engineering and Materials Science , Queen Mary University of London , Mile End Road , London E1 4NS , UK . Email: g.sukhorukov@qmul.ac.uk; c Remote Controlled Theranostic Systems Lab , Saratov State University , 83 Astrakhanskaya Street , Saratov 410012 , Russia

## Abstract

Polyelectrolyte multilayer (PEM) microchambers can provide a versatile cargo delivery system enabling rapid, site-specific drug release on demand. However, experimental evidence for their potential benefits in live human cells is scarce. Equally, practical applications often require substance delivery that is geometrically constrained and highly localized. Here, we establish human-cell biocompatibility and on-demand cargo release properties of the PEM or polylactic acid (PLA)-based microchamber arrays fabricated on a patterned film base. We grow human N2A cells (a neuroblastoma cell line widely used for studies of neurotoxicity) on the surface of the patterned microchamber arrays loaded with either a fluorescent indicator or the ubiquitous excitatory neurotransmitter glutamate. The differentiating human N2A cells show no detrimental effects on viability when growing on either PEM@PLA or PLA-based arrays for up to ten days *in vitro*. Firstly, we use two-photon (2P) excitation with femtosecond laser pulses to open individual microchambers in a controlled way while monitoring release and diffusion of the fluorescent cargo (rhodamine or FITC fluorescent dye). Secondly, we document the increases in intracellular Ca^2+^ in local N2A cells in response to the laser-triggered glutamate release from individual microchambers. The functional cell response is site-specific and reproducible on demand and could be replicated by applying glutamate to the cells using a pressurised micropipette. Time-resolved fluorescence imaging confirms the physiological range of the glutamate-evoked intracellular Ca^2+^ dynamics in the differentiating N2A cells. Our data indicate that the nano-engineering design of the fabricated PEM or PLA-based patterned microchamber arrays could provide a biologically safe and efficient tool for targeted, geometrically constrained drug delivery.

## Abstract 2

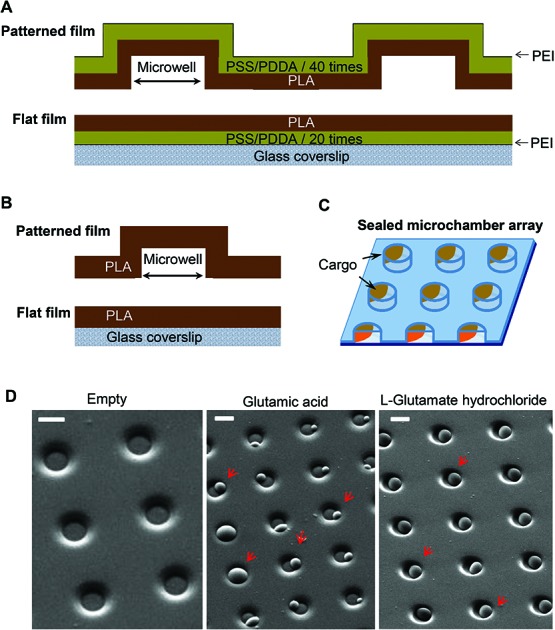
Polyelectrolyte multilayer (PEM) microchambers can provide a versatile cargo delivery system enabling rapid, site-specific drug release on demand.

## Introduction

Over the past decade, various nano-engineering designs have been explored for their capability for micro-packaging, targeted delivery, and controlled release of bioactive compounds. Among such systems, the most prominent were nanoparticles and microcapsules,^1^–^3^ hydrogels,^4^ and nanocomposite films fabricated with the polyelectrolyte-based multilayer (PEM) assembly using the layer-by-layer (LbL) technique.^5^,^6^ Nanostructured PEMs combine multiple functionalities to provide control over cargo release: their shell permeability could be changed by external triggering using either an altered environment (pH, osmolarity) or a physical stimulus (light, temperature, or ultrasound).^7^–^10^ A broad range of functional properties, together with a relatively small size of capsules (nano- to micrometer range), ultimately provided such cargo systems with numerous benefits, prompting growing interest towards translating fundamental studies into practical implementations.^3^,^8^,^11^,^12^ Whilst successful encapsulation has been implemented for a wide variety of substances,^13^ microscopic targeting of cargo release along predetermined space trajectories or geometric patterns has remained elusive. This targeting could be particularly important, for instance, for guiding cell growth or dealing with geometrically defined tissue areas.

Recent advances in PEM films fabricated on patterned surfaces appear to overcome the issue of capsule scattering, by enabling an array of micro-wells or microchambers of predetermined size and spatial distribution.^5^,^6^,^14^ Composed as LbL-assembled PEM films, this novel strategy of housing bioactive compounds combines all functionalities of PEM nanostructures with having a layer of hydrophobic polymer such as polylactic acid (PLA) to increase load capacity. The efficient entrapment and storage of various compounds in PEM microchambers have recently been demonstrated,^15^,^16^ including the use of wall nanocomposites sensitive to external triggering for payload release.^14^,^17^ Clearly, the predetermined patterns of microchambers could provide an opportunity to control the localised release of active compounds in space, time, and dosage. Films containing microchambers could also be deposited as a microscopic coating on the surfaces of stents or implants. It would seem therefore important to understand whether and how this delivery system can be used in the conditions of live cells and their networks.

To address this issue, we grew human N2A cells differentiating to neuronal phenotype on the surface of either PEM@polylactic acid (PLA)-based or PLA-based films to test biocompatibility (neurotoxicity) and safe preservation of low molecular weight substances as the cargo inside the square grid distributed microchambers for up to two weeks *in vitro*. We probed the efficient release of the cargo, the excitatory neurotransmitter glutamate, using a focused laser beam and established the glutamate-induced physiological effects on the adjacent N2A cells. Our results provide evidence for the safe and efficient use of the fabricated microchamber arrays for a pharmacological action on human cells.

## Methods

### Fabrication of microchamber arrays

**Fig. 1:**
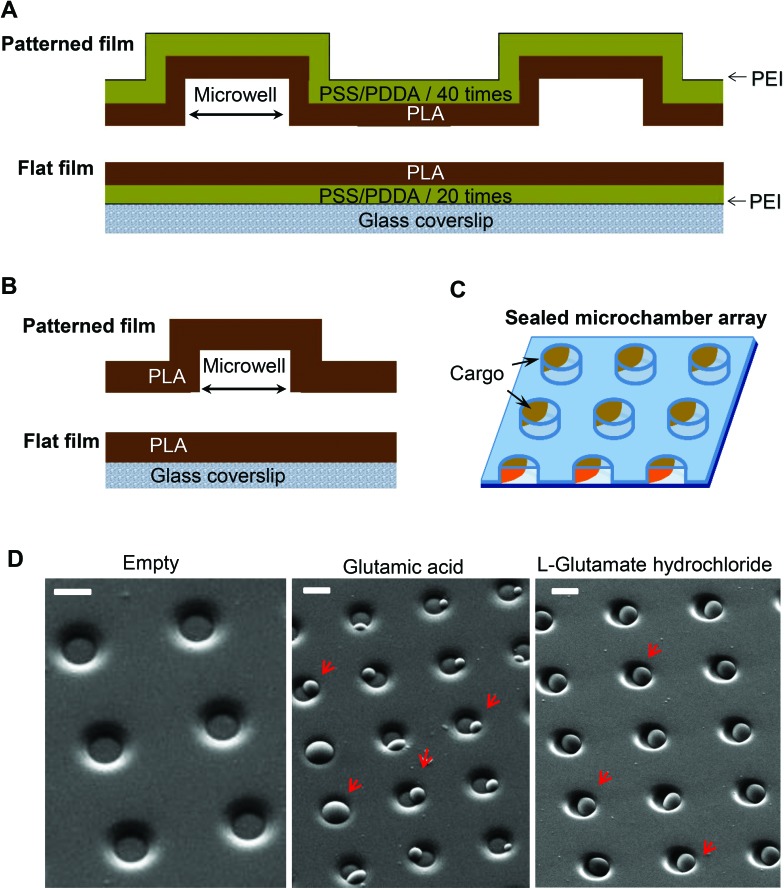
Fabrication of polymer microchamber arrays containing low molecular weight substances as a cargo payload. (A) Fabrication of the PEM@PLA nanocomposite assembly: two films, patterned and flat, each printed separately, before sealing together to compose polymer microchambers. (B) For the PLA-based microchamber arrays, the fabrication process includes production of the patterned film with the microchambers and the flat film on a glass, which are then sealed together. (C) A schematic depicting the sealed microchamber array, with a cargo payload within the microchambers. (D) SEM images of the microchambers (before sealing) for the various types of the fabricated polymer arrays: empty microchambers (no cargo; left image) and microchambers filled with glutamic acid (at the middle) or l-glutamate hydrochloride (right image). Red arrows show a cargo crystal per microchamber. Scale bars, 10 μm.

Patterned microchamber array production was performed as detailed in our previous studies,^6^,^14^,^15^,^17^,^18^ with some modifications. Generally, a patterned microchamber array was composed of two nanocomposite films (Fig. 1A and B), each printed separately and joined after loading a cargo (Fig. 1C). The first film represents a patterned layer containing micro-wells, while the second is flat, without wells, made of the same composite materials.

#### Patterned PDMS stamps

To fabricate patterned films with micro-wells, poly(dimethylsiloxane) (PDMS) stamps were used (Sylgard 184, Dow-Corning, Midland, USA). PDMS stamps were printed out from silicon masters with micropillars. For this, a mixture of a pre-polymer and a curing agent was prepared in a ratio of 10 : 1 and poured on silicon masters, then degassed in a vacuum for 30 min and cured at 70 °C for 3 h. The silicon master was prepared by conventional photolithography. The fabricated micropillars had a cylindrical shape of 10 μm in diameter and 5 μm height.

#### PEM@PLA and PLA production

Different types of microchamber arrays were fabricated, variable by the nanocomposite assembly (PEM@PLA and PLA) and/or the cargo payload (glutamate and its analogue, fluorescent dyes, or empty ones (no payload)), for further functional testing of either array. PEM films were fabricated with the LbL assembly method,^5^,^6^ using a dip-coating robot. Before starting, both PDMS stamp and cover glass (for a flat film production) were submerged in a positively charged poly(ethylenimine) (PEI, Sigma, St Louis, USA) solution for 10 min, followed by washing with ddH_2_O 3 times for 1 min each. Subsequently, two opposite-charged polyelectrolytes, poly(sodium 4-styrenesulfonate) (PSS, Sigma, St Louis, USA) and poly(diallyldimethylammonium chloride) (PDDA, Sigma, St Louis, USA), were layered with the same procedure. Either compound was used at the concentration of 2 g L^–1^ (in 0.5 M NaCl) for each layer. In total, 40 layers of PSS/PDDA were applied on the PDMS stamp and 20 layers of PSS/PDDA on the cover glass. For hydrophobization, the PEM films were immersed in 0.5% polylactic acid (PLA, 3 mm nominal granule size, Sigma, St Louis, USA) for 3 s and dried afterwards (typically, 10 min in air) (Fig. 1A). For fabrication of the PLA-based films, the procedure includes the immersion of both the PDMS stamp and the cover glass in a 0.75% PLA solution for 3 s, followed by drying (Fig. 1B).

#### Cargo loading and microchamber sealing

Several water-soluble, low molecular weight substances were loaded inside the microchambers, including fluorescent dyes (FITC, rhodamine) and the most common excitatory neurotransmitter glutamate (glutamic acid or its pharmacological analogue, l-glutamic acid hydrochloride). Each compound was diluted at a required concentration (near its maximal solubility) and applied onto the surface of the patterned film to fill micro-wells. The compound concentration was as follows: 0.5 μg ml^–1^ for rhodamine and FITC (Sigma, St Louis, USA), 10 μg ml^–1^ for glutamic acid, and 300 μg ml^–1^ for l-glutamic acid hydrochloride (Sigma, St Louis, USA). After filling the micro-wells with the solution, the film was allowed to dry until cargo crystals were formed (Fig. 1D). Finally, the patterned microfilm with the crystals inside the microchambers was sealed to a flat film with a pressure (–2 kg cm^–1^) to make one single unit (Fig. 1C).

To optimise filing of the microchambers, l-glutamic acid hydrochloride (diluted at 300 μg ml^–1^ in ddH_2_O) was applied onto the patterned film surface in 200–300 μl volume batches to fill all microchambers across a sample; the remaining medium was then carefully removed from the film surface (a process facilitated by the hydrophobic PLA surface). As a result, large crystals of l-glutamic acid hydrochloride (clearly visible at a magnification of ×20 and above) were filling the microchambers homogeneously across the sample and, importantly, were of a similar size between the microchambers, as evidenced by SEM images (Fig. 1D). Such a controlled cargo payload for individual microchambers is essential for further functional testing on live cells. The estimated amount of l-glutamic acid hydrochloride was ∼20 pg per microchamber.

### Scanning electron microscopy (SEM)

Scanning electron microscopy (SEM, FEI, Inspect-F) was used to visualize the microchamber morphology at different steps through the fabrication procedure (payload, sealing) and after opening the microchamber(s) to ensure appropriate samples. SEM was carried out using an accelerating voltage of 10 kV, a spot size of 3.5 nm, and a working distance of approximately 10 mm.

### Cell cultures

Human N2A cell line, a gift from the Prof. Stephen Hart's group (UCL, UK), was cultured in Dulbecco's modified Eagle medium (DMEM, Invitrogen, Carlsbad, CA, USA), containing high glucose and 2 mM l-glutamine, supplemented with 10% fetal bovine serum (FBS), 2% penicillin–streptomycin, and 1% non-essential amino acids at 37 °C (5% CO_2_). The cells were harvested using 0.05% trypsin–EDTA (Gibco, USA) for 5–10 min, washed and plated on the surface of microchamber arrays placed individually in a 5 cm^2^ Petri dish. The fabricated microchamber arrays were pre-treated with UV light for at least 2 h prior to plating the cells. For differentiation of the N2A cells to neuronal phenotype, the culturing medium was low serum DMEM (2% FBS instead of 10%). The differentiating N2A cells were maintained on the microchamber array samples until used. For each type of the microchamber arrays, there were at least three independent cell preparations tested.

### Cell viability assessment

To monitor possible concomitant toxic effects of the microchamber arrays, we compared the density of the N2A cells growing on the surface of the PEM@PLA- and PLA-based arrays with that of those growing outside the microchamber area, at various time points of cell growth. Healthy cells were counted within the sampling areas chosen in a quasi-random fashion in the middle of the microchamber arrays, and outside the microchamber area (close to the film edge). Cell density was calculated as the number of viable cells per mm^2^. An additional control group included N2A cells plated on glass coverslips, with no polymer (PLA) present. The other functional indicator of cell viability was the total length of cell neurites per cell. To this end, cell images (sampled as above) were analysed with NeuronJ (ImageJ, NIH).

### Opening of individual microchambers with a femtosecond pulse laser

To optically address individual microchambers, we employed a Femto2D (Femtonics) multi-photon excitation microscope system optically linked to a Ti:Sapphire Mai-Tai femtosecond pulse laser (SpectraPhysics, Newport, ∼220 fs pulses at 80 MHz). The two key advantages of two-photon (2P) excitation (or absorption) with near-infrared light were (i) high penetration (low scattering and absorption) in turbid media compared to visible or UV light and (ii) the 2P excitation effect within a thin (∼1 μm) focal layer only. Another important advantage of femtosecond-pulse excitation in live tissues was that, despite a very high intensity delivered during the pulse, the time-average wattage delivered to the specimen was fully compatible with live function. Indeed, the range of laser power used here was similar to that routinely used by us and others for cell imaging in acute brain slices and in live animals.^19^–^21^

The empirically determined optimal wavelength was 740 nm (opening of the PEM@PLA films) or 780–790 nm (opening of the PLA-based microchambers). For microchamber opening, the laser beam was focused on the top of the microchamber cap (typically, within the cap edges), at a power of 26 mW over 3–5 ms. We ensured that no neighbouring cells were affected by the high-power laser pulses during microchamber opening, as evidenced by the images shown. Otherwise, cell imaging was carried out at a power under the objective of <3 mW, as detailed below. We found that the two protocols for microchamber formation, PEM@PLA and PLA, work equally well for cargo (glutamate) loading and for laser opening. Thus, the presence of the precursor PEM film did not appear to have an effect on the microchamber functionality, which was defined largely by the terminated PLA layer.

#### Two-photon (2P) excitation fluorescence imaging

Cells were bolus loaded with the cell-permeable Ca^2+^ indicator Oregon Green BAPTA-1 (OGB-1 AM; 5 μM, Invitrogen) in the presence of Pluronic F-127 (0.02%, Invitrogen) by incubation for 30 minutes at 37 °C. After loading, the cells were washed for approximately 30 minutes for de-etherification of the dye. A sample was placed in a recording chamber mounted on the stage of an Olympus BX51WI upright microscope (Olympus, Japan) equipped with an XLPlanN 25×/1.05 objective coupled to an infrared DIC imaging system. Imaging was carried out using a Femto2D (Femtonics, Budapest, Hungary) system optically linked to a Ti:Sapphire Mai-Tai femtosecond pulse laser (SpectraPhysics, Newport), and excitation at *λ*2Px = 800 nm optimized for OGB-1. To minimize contaminating fluorescence (PEM/PLA autofluorescence), 2P excitation fluorescence images were taken from a thin focal excitation plane (∼1 μm) within individual cells, which was held unchanged throughout the experiment (prior to and following opening a microchamber). For the time-lapse changes in OGB-1 fluorescence, images were collected using 512 × 512 pixel frames in the stream acquisition mode acquired in 20 s time sections for typically 1 minute before opening a microchamber (baseline) and for 2.5–5 min immediately after the microchamber opening. Changes in [Ca^2+^]_i_ were expressed as the changes in OGB-1 fluorescence at the maximum of the fluorescent signal over the baseline (Δ*F*/*F*_0_), as detailed previously.^22^

The sequence of laser-triggered microchamber opening (pulse 3–5 ms, 1–2 times; *λ* = 790 nm), including focal adjustment and fast scanning in line mode to identify opening, took typically ∼30 s. Recordings were carried out in a HEPES-based medium containing (in mM) 135 NaCl, 5 KCl, 2 CaCl_2_, 2 MgCl_2_, 10 HEPES, 10 glucose (pH 7.4; 290–300 mOsm). In a separate set of experiments, glutamate (5 μM) was locally applied to the cells *via* a fabricated glass micropipette (∼1 μm inner diameter of the tip) to provide a direct comparison of the effects between the triggered release of cargo from the microchambers and the locally applied agonist. To enable a brief, localised agonist application (200–400 ms duration), a pressurised micropipette was connected to the two-channel PDES-02DX pneumatic microejector (npi electronic GmbH) with compressed nitrogen. The fluorescent tracer Alexa Fluor-594 (100 μM) was added into the pipette to visualize the area of the agonist spread.

### Time-resolved fluorescence lifetime imaging (FLIM)

In the context of intracellular Ca^2+^ monitoring, fluorescence lifetime imaging (FLIM) can provide readouts insensitive to concomitant fluctuations in focus or in dye concentration.^21^,^23^,^24^ FLIM was conducted using the in-house system based on the Femto2D microscope equipped with a Becker and Hickl FLIM detector (Femtonics, Budapest), as described earlier.^21^,^23^ The FLIM duty cycle was driven by an 80 MHz infrared pulsed laser (SpectraPhysics, Newport Mai-Tai BB). Fluorescence images were acquired at 2 frames per second and stored as a data tensor representing *x-y* pixel images with a distribution of the nanosecond delay time (*t*) of photons at each pixel over the frame duration (*T*). Average image acquisition times were 240–300 s (maximum frame laser exposure time per acquisition trial of <6 s to minimize phototoxic damage), depending on the total photon count; the maximum photon count rate was on average <10^5^ s^–1^ which is well below the effect of photon pile-up. To monitor changes in [Ca^2+^], we minimized the sampled image area with various digital zooms to approximately 70 × 70 μm (*x*, *y*). Because of the relatively low photon counts per pixel, the photon data were accumulated over the selected ROI from the (*t*, *x*, *y*, *T*) tensor into the (*t*, *T*) matrix before the data analysis was used to estimate [Ca^2+^]. To confirm changes in the OGB-1 fluorescence intensity, we also calculated the intensity from FLIM data, by integration of the photon counting data (non-normalized) at the same ROI (Fig. 6A).

### Statistical analysis

All data are presented as mean ± standard error of the mean, with *n* referring typically to the number of cells analyzed within the experimental groups. For the cell viability analysis, n refers to the number of different areas/samples where the cell density was assessed or indicates the number of neurites measured for their length. To determine statistical difference between the experimental groups, Student's *t*-test (two-tailed paired or unpaired) was used where appropriate. A *p* value of less than 0.05 was considered as statistically significant.

## Results and discussion

### Biocompatibility and cargo preservation

**Fig. 2:**
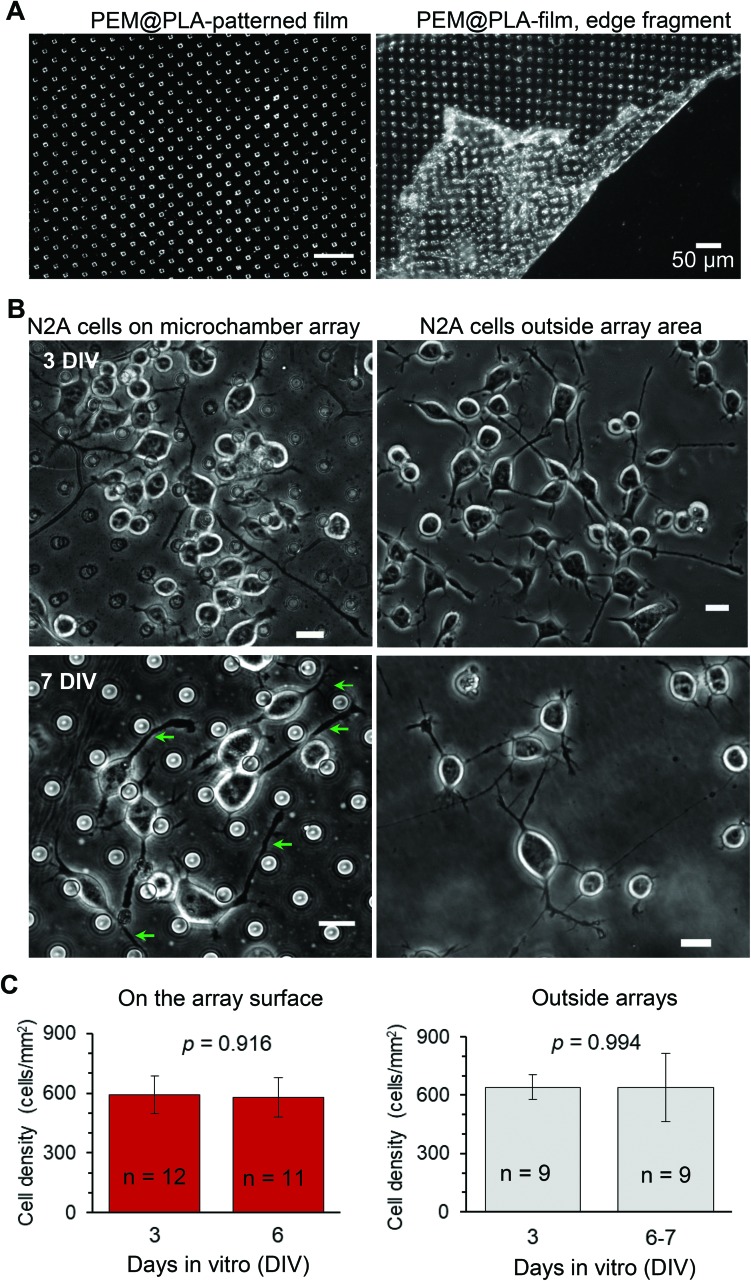
Biocompatibility and cargo preservation within the microchambers: the fabricated PEM@PLA microchamber arrays. (A) Transmitted light images of PEM@PLA-patterned microchamber arrays, central area (left) and towards the edge, depicting the detached PEM film (right). Scale bars, 50 μm. (B) Representative images of the differentiating N2A cells growing on the surface of the microchambers loaded with glutamic acid (left row) or outside the microchamber area (right row) at different time points after cell plating. Green arrows, axon-like processes in the N2A cells of neuronal phenotype. Scale bars, 15 μm. (C) Statistical summary, cell density (mean ± s.e.m.) on top of the microchambers loaded with glutamate (left) and outside the microchambers (right), at three days *in vitro* (DIV3) and DIV6–7; *n*, number of sampled areas; *p*, two-tailed, unpaired *t*-test.

**Fig. 3:**
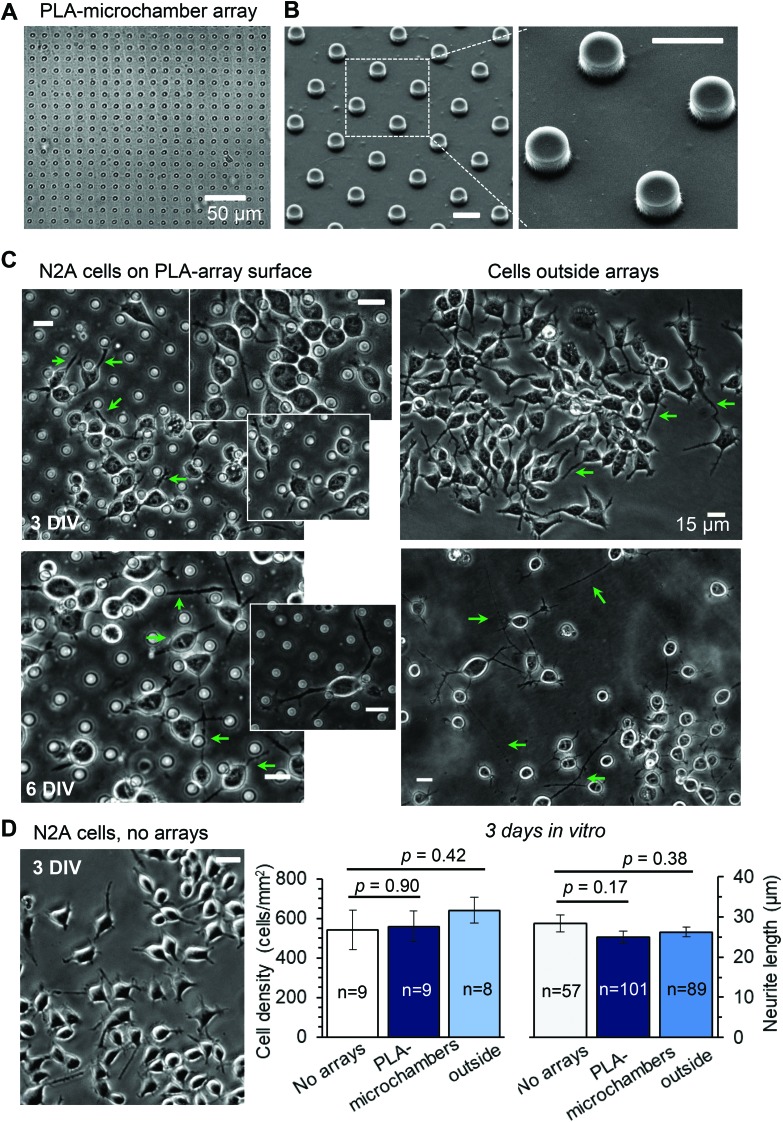
The differentiating N2A cells show no signs of excitotoxicity when growing on the PLA-based microchamber arrays with the glutamate payload. (A–B) The representative transmitted light image (A) and SEM images (B) of the PLA-based microchamber array after sealing the microchambers with loaded glutamic acid inside. Scale bars in B, 10 μm. (C) Representative snapshots of the differentiating N2A cells growing on the surface of the PLA-based microchambers loaded with glutamate (left panels) and outside the microchamber area (right panels) at different times after cell plating. Green arrows, axon-like processes in the N2A cells of neuronal phenotype. Scale bars, 15 μm. (D)A snapshot of the differentiating N2A cells on a glass coverslip at DIV3. Plots, statistical summary, cell density (left) and neurite length (right, mean ± s.e.m.) for the cells growing on the coverslips, the PLA-based microchambers containing glutamate, in the area outside microchambers; *n*, number of sampled areas (left) or measured neurites (right); *p*, two-tailed, unpaired *t*-test.

The neuroblastoma cell line (N2A) was selected to provide fast cell growth, with the cells differentiating to neuronal phenotype.^25^–^28^ We placed the stamped arrays of microchambers (Fig. 1) filled with either glutamate or fluorescent indicator, or left empty, in the cell culture medium, and subsequently plated the N2A cells to grow on the array surface, or entirely outside the arrays, during 7–10 days (Methods). The two array types tested were PEM@PLA (Fig. 2) and PLA (Fig. 3).

24 h post plating, the N2A cells developed typical axon-like processes and extended neurites (*e.g.*, Fig. 2B and 3B). To assess cell viability, we monitored the length of such processes, along with the cell density, throughout the preparation, for up to ten days *in vitro* (Methods). During that time, cell processes could reach up to 300 μm in length, suggesting no gross detrimental effects of having the fabricated arrays nearby. On a finer scale, we found no difference in the cell viability among the samples containing microchambers, with or without loaded glutamate, inside or outside the chamber areas (or with no polymer material present), for both array fabrication types, over 3–7 days *in vitro* (Fig. 2C and 3C). This observation is consistent with the reported biocompatibility of the constituent polymers, PSS and PLA.^29^,^30^ Also, given that the differentiating N2A cells are highly sensitive to low glutamate levels,^25^,^31^ this result also suggests the microchamber wall stability in preventing cargo escape.

### Localised cargo release by optical targeting of individual microchambers

**Fig. 4:**
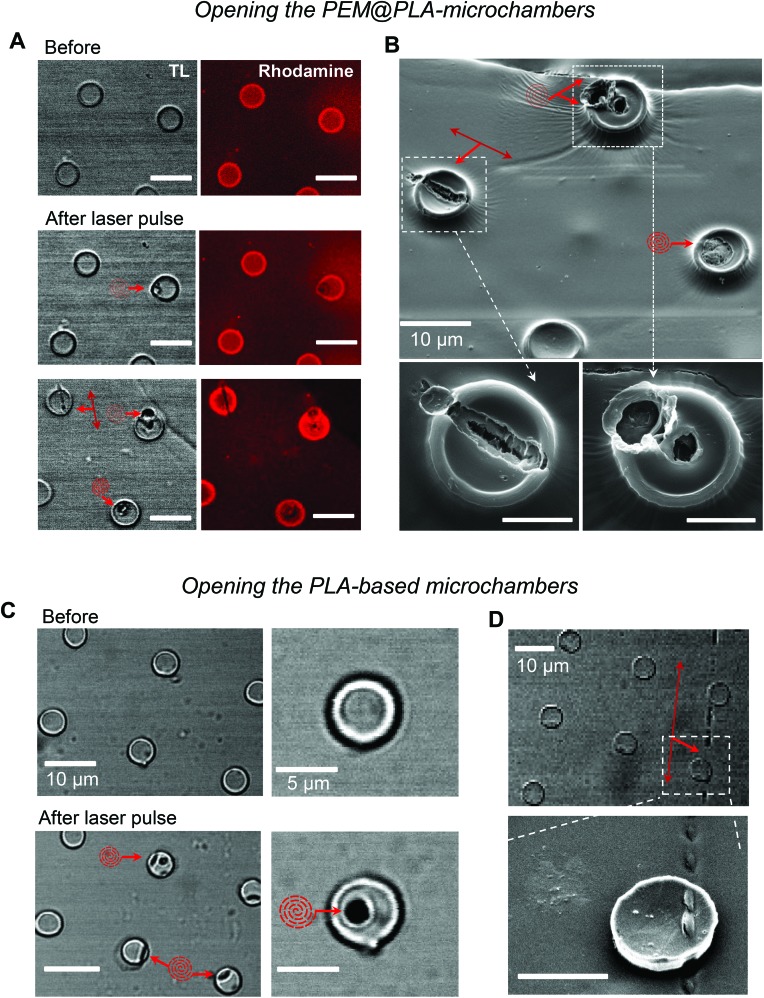
Optical targeting of individual microchambers with a femtosecond infrared pulse. (A) Images of the PEM@PLA-microchambers (transmitted light, TL, and rhodamine fluorescence channels, *λ*2Px = 800 nm) before and after applying a focused laser beam (*λ* = 740 nm) for opening the selected microchambers, using various trajectories (line or spiral) as indicated. Red arrows depict the pattern applied to open a microchamber. Scale bars, 10 μm. (B) SEM images of the microchamber morphology after opening with a femtosecond pulse laser. Images are taken from the same area as for the bottom row in A (microchambers match), showing successful opening with various patterns of a laser beam applied to the microchamber surface. Red arrows, patterns used for opening. Scale bars for bottom images, 5 μm. (C) Transmitted light images of the PLA-based microchamber arrays before and after applying a femtosecond pulse laser for microchamber opening (spiral trajectories, 3 μm wide). (D) SEM images of the opened microchamber, indicated in the top snapshot, using a line segment laser trajectory. Scale bars, 10 μm.

**Fig. 5:**
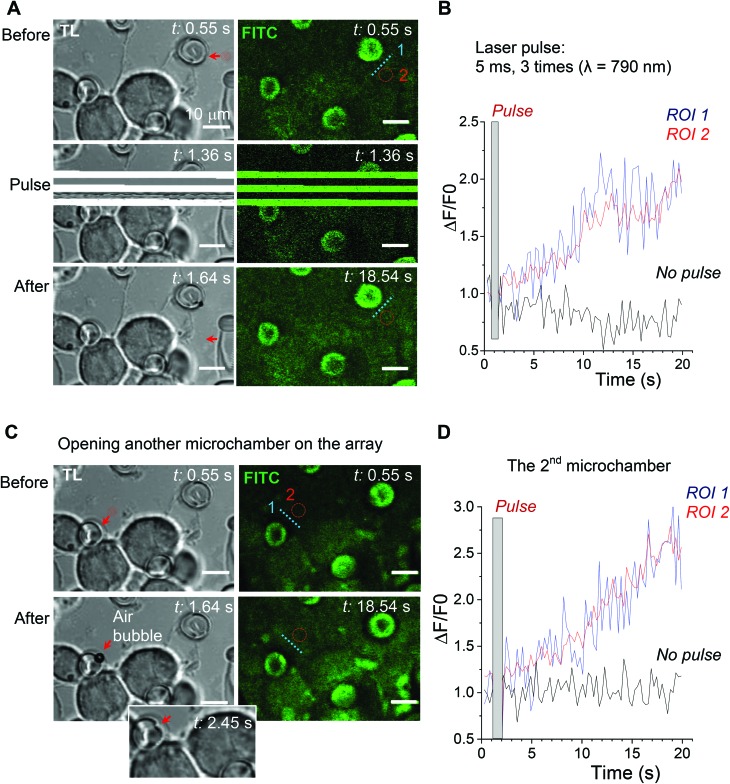
Release of the fluorescent cargo from individual microchambers using optical targeting. (A) The PEM@PLA microchamber array with the FITC payload in microchambers (transmitted light, TL, and FITC fluorescence channels; *λ*2Px = 800 nm), before (top), during (middle), and after (bottom) optical targeting of a microchamber with a femtosecond laser beam (*λ* = 790 nm; 26 mW, 3 consequent pulses, for 5 ms each). An air bubble formed upon the laser pulse confirms the microchamber opening. Scale bars, 10 μm. (B) Time course of changes in FITC fluorescence over regions of interest (ROIs), as indicated in A. (C) The same PEM@PLA microchamber array as for A and B (FITC payload) while targeting another microchamber. Air bubble formation confirms the microchamber opening, followed by visualization of the opened (disfigured) microchamber. Scale bars, 10 μm. (D) Time course of changes in FITC fluorescence over various ROIs, as indicated in C.

**Fig. 6:**
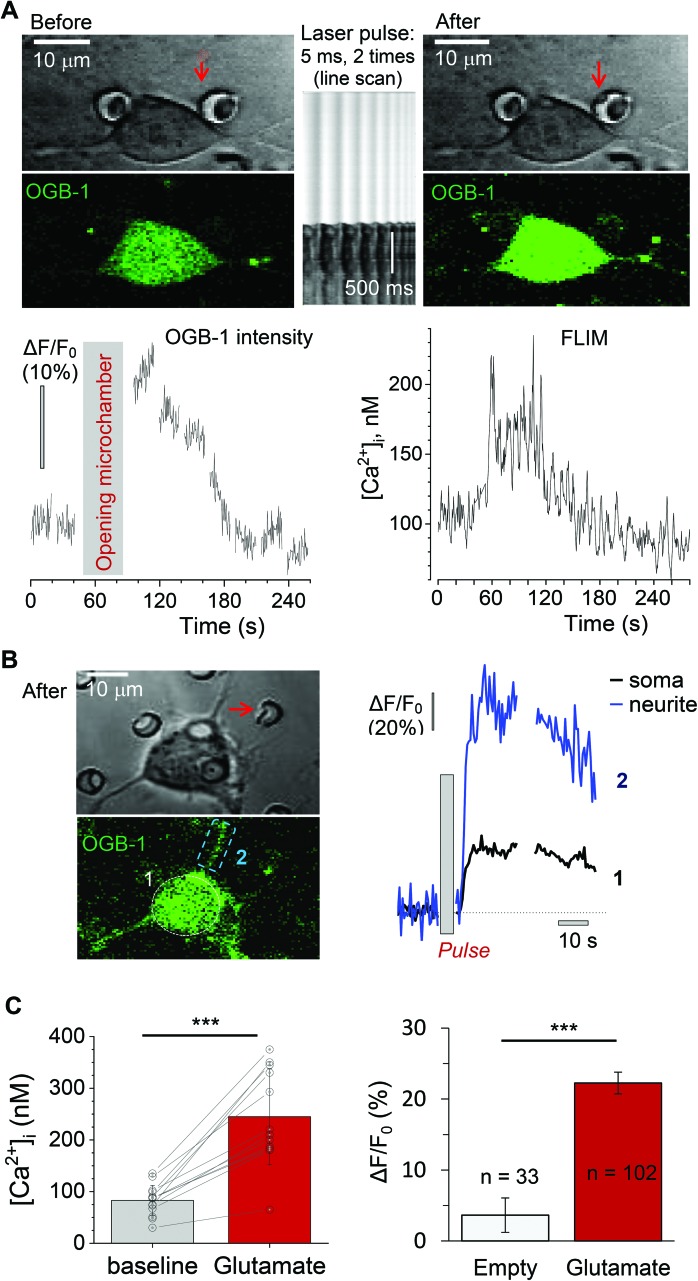
Monitoring the intracellular Ca^2+^ rise in the differentiating N2A cells upon the triggered release of glutamate from the microchambers. (A) An example of a differentiated N2A cell grown on the PLA-based microchamber array loaded with glutamate before (left) and after (right) local microchamber opening (dotted line). Upper row, transmitted light images; bottom row images, the OGB-1 fluorescence channel (*λ*2Px = 800 nm). Middle panel, line scan across the dotted line as shown in the left image, confirming the microchamber opening with a femtosecond pulse laser (*λ* = 790 nm; 26 mW); scale bars, 10 μm. Plots, time courses of the OGB-1 fluorescence intensity signal Δ*F*/*F* (left) and of [Ca^2+^]_i_ (FLIM readout, right), during chamber opening, as indicated. (B) Left panels: A DIC image (top) and OGB-1 channel snapshot (bottom); dotted areas, ROIs 1 and 2, for the soma and neurites, respectively; red arrow, the targeted microchamber. Plots: time course of the Ca^2+^ rise in the cell soma (ROI 1) and neurites (ROI 2), during the glutamate release from a microchamber, as indicated. (C) Statistical summary. Left, [Ca^2+^]_i_ levels in the N2A cells before and following microchamber opening (FLIM readout, *n* = 14 cells). Right, relative changes in OGB-1 fluorescence intensity in response to optical targeting of the PLA-based microchambers with no payload (empty) and loaded with glutamate (red); *n*, the total N2A cells analysed across four independent experiments for each type of fabricated arrays. ***, *p* < 0.0001 (two-tailed, paired and unpaired *t*-test for left and right plots, respectively).

Next, we used laser pulses to open individual microchambers at selected sites (Methods). We thus tested varied beam trajectories (line, spiral, point) to open individual microchambers across the polymer surface, also exploring varied exposure areas (from 1 to 5 μm; Fig. 4). The successful opening of the targeted microchambers, with fully intact neighbours, was confirmed with SEM (Fig. 4B and D). Importantly, cultured N2A cells nearby remained unperturbed (Fig. 5 and 6). We next tested whether the payload inside the microchambers remained intact after loading and optical addressing (laser beam opening). First, we used a fluorescent dye, either rhodamine or FITC, as the cargo. The presence of fluorescent emission inside the microchambers was confirmed using 2P excitation imaging at the plane of microchambers, for either loaded dye (Fig. 4A for rhodamine and Fig. 5 for FITC). We were also able to visualise the release of the loaded dye upon microchamber opening, using the time-lapse imaging: a prompt and robust rise in the FITC-mediated fluorescence next to the opened microchamber could be detected over the regions of interest (ROIs, indicated in Fig. 5A and C) shortly after the laser pulse (Fig. 5B and D). Appearance of an air bubble (transmitted light channel) immediately after the pulse indicated microchamber opening (Fig. 5A and C), as reported in previous studies.^15^ Control experiments confirmed that there was no rise in FITC fluorescence (no payload release) over the same ROI when no laser pulse was applied (Fig. 5B and D) or when repeatedly targeting the opened microchamber. Subsequent targeting of the intact microchambers triggered a similar rise in local FITC fluorescence over the neighbour area (Fig. 5C and D), confirming the repeatable effect on demand.

### Laser-triggered glutamate release from individual microchambers mobilises intracellular Ca^2+^ in local N2A cells

Finally, we evaluated functional effects of the triggered cargo release on live cells. To this end, we used glutamate-loaded microchambers: in N2A cells, glutamate induces a prominent inward current through the Ca^2+^-permeable *N*-methyl-d-aspartate (NMDA) receptors.^32^ We therefore employed 2P excitation imaging of the Ca^2+^ indicator Oregon Green BAPTA-1 (OGB-1) bulk-loaded into the differentiated N2A cells growing (3–6 days) on the microchamber arrays filled with glutamate (l-glutamic acid hydrochloride, Methods; Fig. 1D). Imaging was carried out in continuous frame-scanning mode (∼500 ms per frame), before opening a microchamber (baseline) and immediately afterwards (up to several minutes; Fig. 6A). The chamber opening phase, including line scan (top middle image in Fig. 6A) and focal adjustment, took ∼30 s (Methods). In the proximity of the glutamate-filled microchambers, we could detect clear Ca^2+^ rises inside the cells following local microchamber opening. The rises were transient and declined back close to the resting baseline level within minutes of microchamber opening (Fig. 6A, bottom). The time course of the Ca^2+^ rise detected in the N2A cell soma and neurites displayed comparable kinetic profiles (Fig. 6B). The functional effect was measured in 102 cells growing on top of the glutamate-loaded microchambers to be tested for opening (about 22 microchambers in total) across 4 PLA-microchamber array samples (*n* = 4 independent cell culture preparations; Fig. 6C). In contrast, there were no detectable changes in Ca^2+^ dynamics in the N2A cells growing on the surface of the PLA arrays containing empty microchambers (no payload), upon their opening (*n* = 33; *p* < 0.001 compared with the group of cells on the glutamate-containing microchambers; Fig. 6C).

To verify that the observed Ca^2+^ transients were within the physiological range and not affected by focus fluctuations, cell deterioration, or concomitant laser pulse effects, we used a FLIM method to document the ‘absolute’ intracellular Ca^2+^ concentration ([Ca^2+^]_i_) dynamics, as shown earlier.^21^,^23^ Thus, we obtained the direct readout of the intracellular Ca^2+^ concentration ([Ca^2+^]_i_) in the N2A cells before and after opening the microchambers (Fig. 6A bottom). Our data demonstrated a low resting [Ca^2+^]_i_ in the differentiated N2A cells (mean [Ca^2+^]_i_ value: 83 ± 8 nM, *n* = 14 cells; Fig. 6C), a fully relevant physiological level similar to the neuronal [Ca^2+^]_i_ reported *in situ*.^21^,^33^ Glutamate release from the microchambers triggered a rapid [Ca^2+^]_i_ rise, in the 200–400 nm range, in each of the analysed cells (*p* < 0.001; Fig. 6C), which is consistent with the Ca^2+^ dynamics in neurons *in vivo*.^19^

**Fig. 7:**
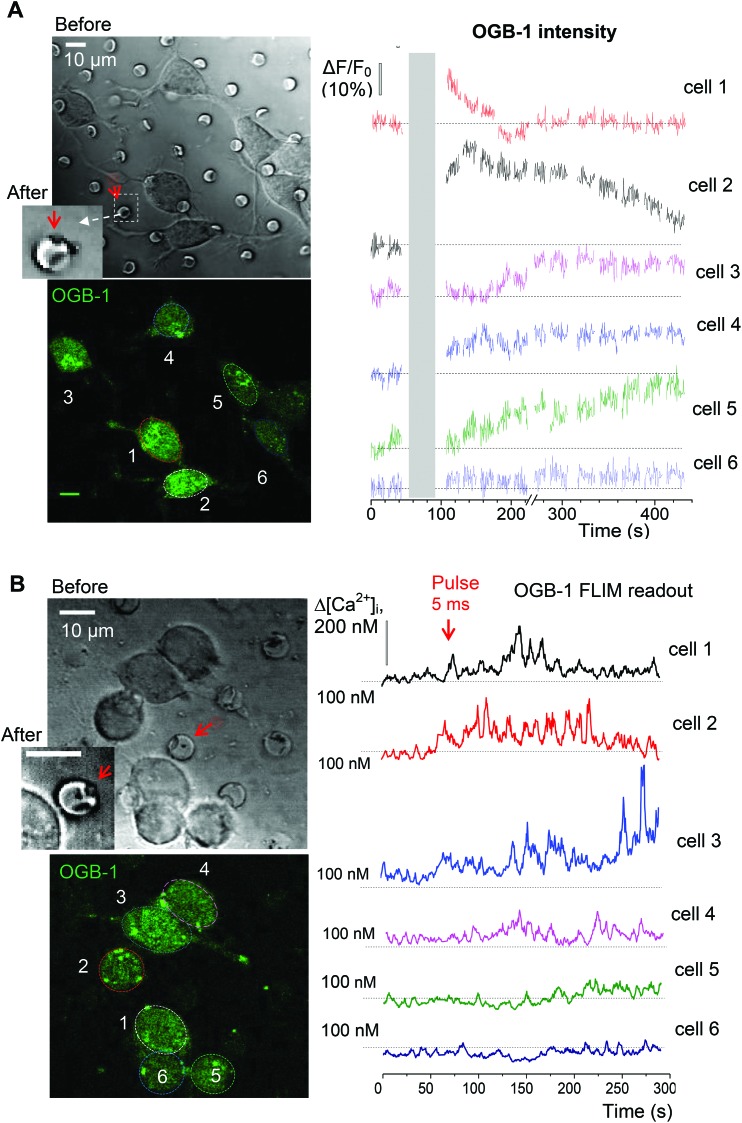
Monitoring [Ca^2+^]_i_ dynamics in the adjacent N2A cells upon the triggered release of glutamate from individual microchambers. (A) Transmitted light (top) and fluorescence images (bottom, OGB-1 fluorescence channel; *λ*2Px = 800 nm) of the differentiating N2A cells growing on the surface of the PLA-based microchambers loaded with glutamate before and after opening the selected microchamber. Red arrow, a microchamber before and after opening. Plots, time course of changes in OGB-1 fluorescence intensity for individual cells as shown in the bottom left image. (B) Experiment as in A, but monitoring [Ca^2+^]_i_ in the N2A cells using the OGB-1 FLIM readout. Scale bars, 10 μm.

**Fig. 8:**
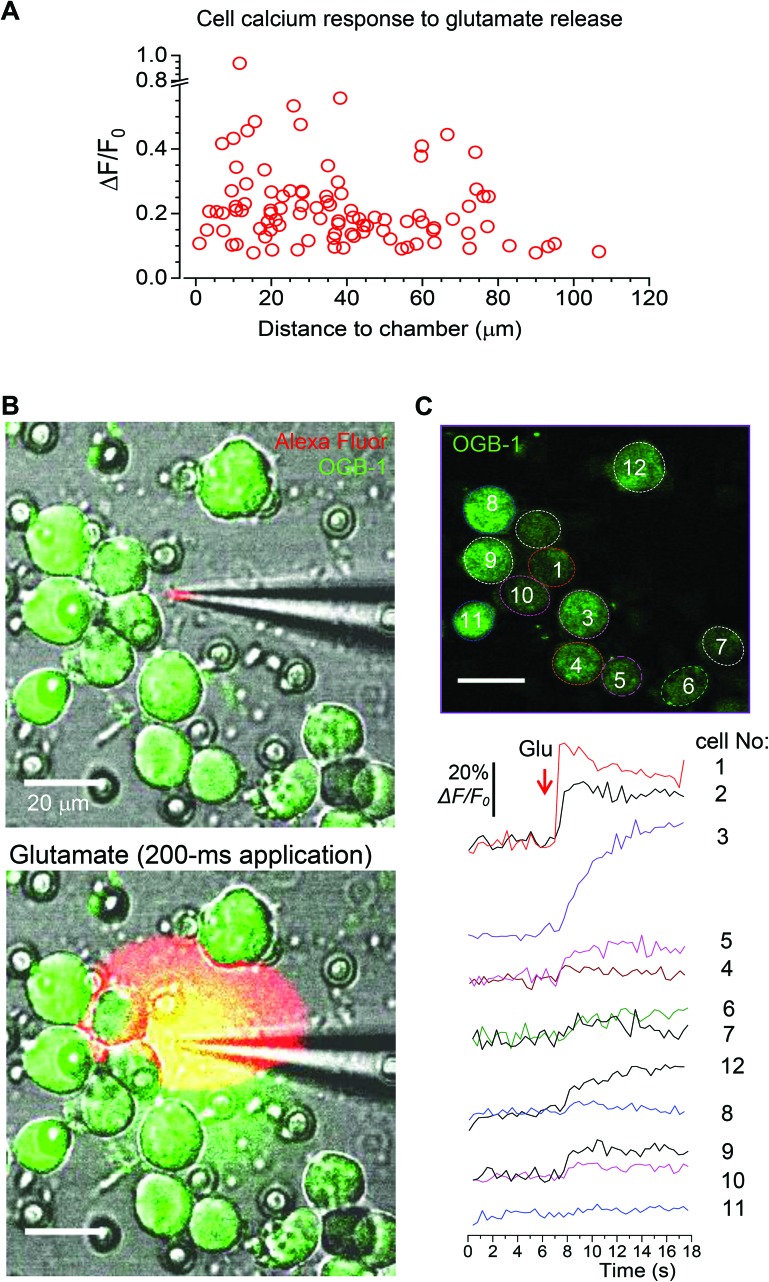
Replicating the functional effect of the glutamate release from the microchambers with a focal agonist application onto the N2A cells. (A) Summary of the Ca^2+^ rise in individual N2A cells (*n* = 102) in response to the triggered glutamate release from the microchambers, plotted against the distance from the targeted microchambers. (B) Images of the differentiating N2A cells growing on the surface of the PLA-based microchamber array before (top) and after (bottom) a direct focal application of glutamate (5 μM, 200 ms duration) through a micropipette positioned in the close proximity of the cells. Images, combined transmitted light, OGB-1 (green), and Alexa Fluor-594 (red) channels (*λ*2Px = 800 nm). (C) The same area as in A, showing the N2A cells at the OGB-1 fluorescence channel (*λ*2Px = 800 nm); regions of interest (ROIs) are shown; red arrow, glutamate puff through the micropipette (dotted lines). Bottom plots, time course of the Ca^2+^ rise over indicated ROI before and after glutamate application (5 μM, 200 ms); red arrow, timing of glutamate puff.

Finally, to assess how far the released glutamate can spread following triggered microchamber opening, we monitored the [Ca^2+^]_i_ dynamics in N2A cells located at different distances from the targeted microchamber. We found transient changes in [Ca^2+^]_i_ (somata) only in cells located at <50 μm from the opened microchamber, across the targeted areas (Fig. 7 and 8A). Again, the functional effect could be detected using the OGB-1 fluorescence readout (Fig. 7A) and FLIM measurements (Fig. 7B). This was fully compatible with the spatial effects of glutamate which was applied *via* a pressurized micropipette (5 μM glutamate, Methods), with the fluorescent control of the puff (Alexa Fluor-594 inside the micropipette; Fig. 8B). These recordings demonstrated a prompt, site-specific [Ca^2+^]_i_ rise in differentiated N2A cells whose dynamics depended on the localization of cells towards the site of agonist release (Fig. 8C). It is noteworthy that, with a suitable adaptation of the microchamber properties, their opening could be, in principle, enacted along a moving trajectory of laser pulses, thus enabling a more comprehensive space–time control of substance release.

## Conclusions

The present study provides experimental evidence indicating that the fabricated polymer microchamber arrays (PEM- and/or PLA-based) could be an efficient drug delivery system for the site-specific, geometry-bound targeting of human cells. Human N2A cells of neuronal phenotype, a highly environment-sensitive cell type, showed no detectable toxicity effects when grown on the PEM@PLA- or PLA-based film surfaces over at least ten days. This suggested (a) high biocompatibility of the film material and (b) reliable drug retention (no leakage) over days. One important advantage we observed was the successful optical addressing of individual microchambers with a focused laser beam, providing site targeting on the micron scale, without employing gold or other light-absorbing nanoparticles normally required to facilitate microchamber opening. Thus, having the precursor PEM film did not appear essential in microchamber fabrication, in the context, which should facilitate the fabrication process.

As a proof-of-concept, this study also demonstrates functional effects triggered by cargo release in live human cells of neuronal phenotype. The action was localised and repeatable on demand and could be reproduced by local puff application of the cargo. Further studies will be required to address the operational capability of polymer patterned arrays within organised tissue, *in situ* and *in vivo*, for their potential implementation towards controlled drug delivery throughout the tissue suitable for various therapeutic interventions.

## Author contributions

D.A.R. and G.B.S. conceived the study and its research strategy; O.K. and K.Z. designed imaging experiments; O.K. carried out experiments and data analyses; K.Z. performed FLIM quantification; O.S. and M.G. fabricated polymer microchambers; O.S. performed SEM; all authors contributed to manuscript preparation.

## Conflicts of interest

The authors declare no conflict of interests.
